# Directed evolution of GFP with non-natural amino acids identifies residues for augmenting and photoswitching fluorescence†
†Electronic supplementary information (ESI) available: Detailed experimental methods, supplementary Fig. 1 to 11 and supplementary Tables 1–3. See DOI: 10.1039/c4sc02827a



**DOI:** 10.1039/c4sc02827a

**Published:** 2014-11-07

**Authors:** Samuel C. Reddington, Amy J. Baldwin, Rebecca Thompson, Andrea Brancale, Eric M. Tippmann, D. Dafydd Jones

**Affiliations:** a School of Biosciences , Cardiff University , Cardiff CF10 3AT , UK . Email: jonesdd@cardiff.ac.uk ; Tel: +44 (0)29 20874290; b School of Chemistry , Cardiff University , Cardiff , UK; c School of Pharmacy and Pharmaceutical Sciences , Cardiff University , Cardiff , UK

## Abstract

Genetic code reprogramming allows proteins to sample new chemistry through the defined and targeted introduction of non-natural amino acids (nAAs). Many useful nAAs are derivatives of the natural aromatic amino acid tyrosine, with the *para* OH group replaced with useful but often bulkier substituents. Extending residue sampling by directed evolution identified positions in Green Fluorescent Protein tolerant to aromatic nAAs, including identification of novel sites that modulate fluorescence. Replacement of the buried L44 residue by photosensitive *p*-azidophenylalanine (azF) conferred environmentally sensitive photoswitching. *In silico* modelling of the L44azF dark state provided an insight into the mechanism of action through modulation of the hydrogen bonding network surrounding the chromophore. Targeted mutagenesis of T203 with aromatic nAAs to introduce π-stacking with the chromophore successfully generated red shifted versions of GFP. Incorporation of azF at residue 203 conferred high photosensitivity on sfGFP with even ambient light mediating a functional switch. Thus, engineering proteins with non-natural aromatic amino acids by surveying a wide residue set can introduce new and beneficial properties into a protein through the sampling of non-intuitive mutations. Coupled with retrospective *in silico* modelling, this will facilitate both our understanding of the impact of nAAs on protein structure and function, and future design endeavours.

## Abstract 2

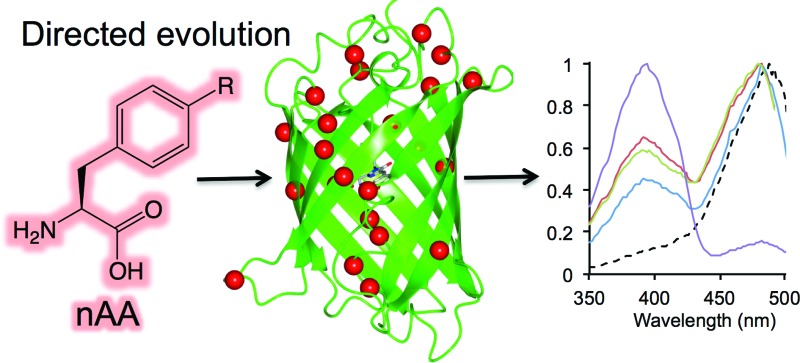
Genetic code reprogramming allows proteins to sample new chemistry through targeted introduction of non-natural amino acids. By combining with random codon replacement, residues traditionally overlooked can be identified as instilling new properties on a target protein.

## Introduction

**Fig. 1:**
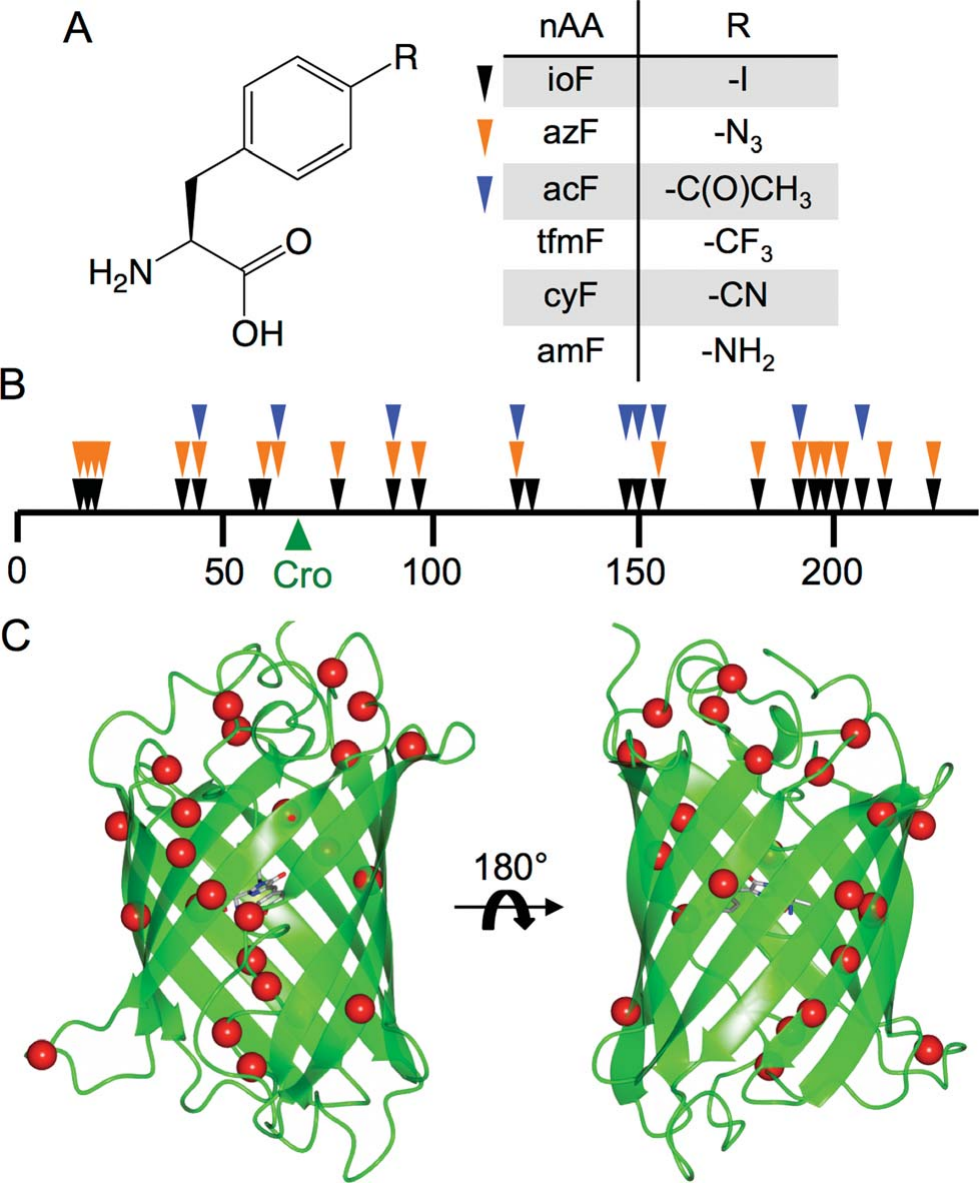
Tolerance of sfGFP to aromatic nAA incorporation. (a) Structure of the aromatic non-natural amino acids used in this study including the 3 aromatic nAAs used in library screening (ioF, azF and acF). (b) Linear map of sfGFP sequence showing sites tolerant to aromatic nAA incorporation as arrows. Each colour represents variants found from screening with three different aromatic nAAs (ioF, black; azF, orange; acF, blue). The position of the sfGFP chromophore (“Cro”) is shown as a green arrow. A more detailed description is provided in ESI Table 1.† (c) 3D structure of sfGFP showing the positions of tolerated aromatic nAA incorporation sites as red spheres.

Reprogramming the genetic code to allow incorporation of non-natural amino acids (nAAs) is a powerful way of engineering proteins by expanding the chemistry sampled (see ref. 1–4 for recent reviews). A wide variety of both aliphatic and aromatic nAAs can now be used to introduce physicochemical properties not normally sampled by the 20 natural amino acids. One of the most developed recombinant methods to incorporate nAAs into proteins *in situ* is amber stop codon (TAG) reprogramming through the use of orthogonal tRNA/aminoacyl-tRNA-synthetase pairs engineered for a specific nAA.^5^ Recently, *E. coli* has been engineered to remove all amber stop codons from the genome and the associated termination factor (RF-1) to truly reprogram TAG for nAA incorporation.^6^ Aromatic nAAs (see Fig. 1 for some examples) based around the natural amino acid tyrosine have proved a particularly fruitful and well-utilised source of new chemistry,^2,7,8^ driven by the use of engineered versions of the versatile tyrosyl-tRNA synthetase.

While rational protein engineering using nAAs is becoming more widespread, it still suffers from the drawbacks of traditional rational site-directed mutagenesis commonly used to implement the approach: predicting the impact of nAA incorporation on protein structure, function and folding. For protein engineering with nAAs to reach its true potential, more information is required on the tolerance of a protein to nAA incorporation and the subsequent impact on the structure–function relationship. This is beginning to be addressed, including with GFP,^9–12^ through more detailed structure–function investigations of site directed mutations. The concept of directed evolution^13–15^ was developed to overcome such predictive problems encountered during traditional protein engineering, and genetic library generation methods have recently emerged for broader nAA sampling.^16,17^ By sampling the whole protein backbone, it is possible to uncover influential residues that would have otherwise been overlooked because, for example, they are distal from the active site or their actions are exerted indirectly on active site residues. Retrospective analysis of such unexpected beneficial mutations can then feedback to the protein design process.

Here we report the use of directed evolution to sample different residues positions and aromatic nAAs to investigate tolerance by and impact on Green Fluorescent Protein. GFP has been the subject of extensive protein engineering endeavours (including by directed evolution) to expand its usefulness as a genetically encoded imaging and reporting agent. Current GFP engineering endeavours focus on generating responsive variants, especially photoswitching for super-resolution imaging.^18,19^ The “superfolder” GFP variant (sfGFP) that is the focus of this work is a fast folding, maturing and highly stable engineered version^21^ of the protein but has no inherent photoswitching behaviour. A library generated by random amber stop codon replacement mutagenesis and one site-directed variant (T203) was screened with different aromatic nAAs to select variants that retained fluorescence so assessing sfGFP's tolerance to nAA incorporation. T203 was targeted, as replacement with aromatic residues is known to influence the fluorescent properties of GFP through π stacking with the chromophore^24^ but was not sampled during selection. A number of variants were identified that had altered spectral properties and displayed photosensitive behaviour, with some nAA incorporation sites hitherto unexplored by traditional mutagenesis. *In silico* modelling was used to investigate the impact of a nAA at residue 44 on GFP structure and thus function, with the aim of facilitating the future design of useful proteins containing new chemistry.

## Results and discussion

### Random TAG codon replacement mutagenesis

The nAAs selected as part of this study are derivatives of phenylalanine (Fig. 1a) with the *para* position substituted with various chemical groups not present in the natural amino acid repertoire. To sample aromatic nAA incorporation across the breadth of sfGFP, a recently developed directed evolution method based on trinucleotide exchange (TriNEx;^20^) (ESI Fig. 1†) was used to introduce TAG codons at random positions throughout the gene. A detailed description of the library construction process is outlined in the ESI.†

The protein appeared tolerant to a variety of aromatic nAAs incorporated at different positions, demonstrating the structural plasticity of sfGFP, even to novel chemical substituents (Fig. 1). Sequence analysis of randomly selected library members that conferred cellular fluorescence when grown in the presence of the aromatic nAAs ioF, azF and acF revealed that all of the variants had in-frame TAG codons distributed throughout the *sfGFP* gene (Fig. 1b; ESI† Table 1†). Identical mutations were observed independently on selection in the presence of different aromatic nAAs, with 5 of the mutation sites observed when screened with all three aromatic nAAs (Fig. 1b).

The majority of tolerated aromatic nAA substitutions involved residues resident in β-strands (60%), in keeping with the predominance of this secondary structure element (comprising ∼50% of residues) in sfGFP (Fig. 1c).^21^ Residues normally buried within the core of sfGFP, which generally comprise aliphatic side chains, were remarkably tolerant to aromatic nAA incorporation despite their bulkier nature and polar *para* substituents; 10 buried residues and 5 partially exposed residues were tolerant to aromatic nAA incorporation (ESI† Table 1†). These include hydrophobic core residues V16, L44, L119, V150, L201 that are close to the chromophore. Three residues in the core helix, P56, W57 and L60, housing the buried chromophore (comprised of G65-Y66-T67) were also found to be tolerant to at least one of the chosen aromatic nAAs (Fig. 1c; ESI† Table 1†).

### Fluorescence properties of aromatic nAA substituted GFP variants

**Fig. 2:**
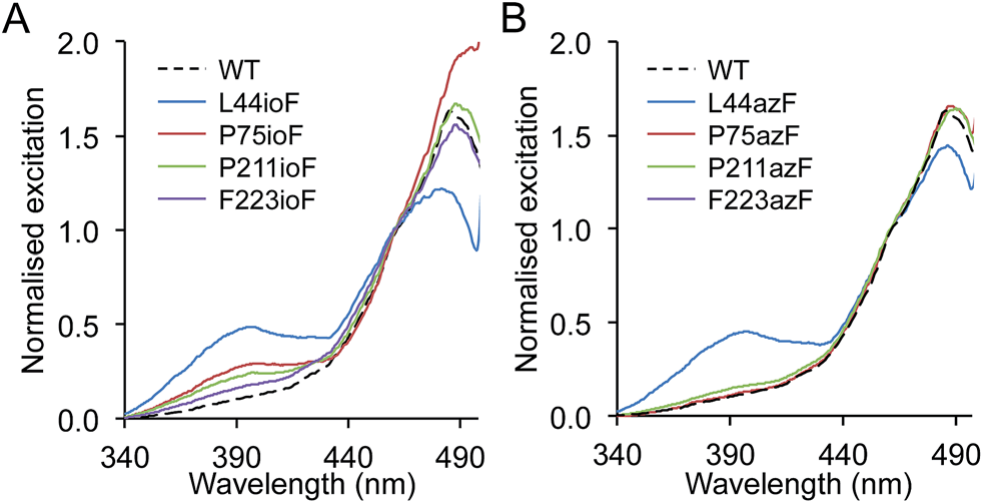
Spectral properties of sfGFP^nAA^ variants. Fluorescence excitation spectra of (a) sfGFP^ioF^ and (b) sfGFP^azF^ variants. Spectra were recorded on cell lysates (soluble fraction) by monitoring emission at 511 nm. Spectra were normalised to a value of 1 at 460 nm.

The chromophore at the heart of all GFP-like fluorescent proteins is very sensitive to its immediate chemical environment, a feature that has been utilised by both nature and protein engineers to generate a broad colour palette and introduce new responsive properties.^18,22^ To assess the impact of aromatic nAA incorporation on sfGFP function, the fluorescence spectra of each unique mutation site observed during the selection phase were investigated using ioF or azF. In most cases, replacement of the native amino acid with ioF or azF had little or no effect on the excitation or emission spectra (ESI† Table 2†) but several mutations did elicit an effect, predominantly on the excitation spectra (Fig. 2). The most significant change observed was the promotion or suppression of the minor excitation peak at ∼400 nm, which reports on the charge state of the phenolic group of the chromophore.^23,24^ In the ground state, the neutral, protonated phenol group excites at ∼400 nm while the anionic, deprotonated form excites at ∼485 nm.

Replacing L44, P75, P211 and F223 with ioF resulted in promotion of the excitation peak at ∼400 nm (Fig. 2a; ESI† Table 2†). The L44ioF variant generated the largest change in the 485 : 400 nm ratio (1 : 0.4) of all the variants. L44 is buried within the core of the protein (ESI† Table 1†) and lies close to the chromophore but does not directly interact with the phenol group. In the case of P75, P211 and F223, it is not clear how ioF is eliciting an effect given their distance, surface exposure and relative positioning with relation to the chromophore but is most likely to be *via* small and propagated changes in the protein structure. However, the effects appear to be specific to the steric bulk of ioF. Replacement of the iodo group with an azido results in only the L44azF retaining a significant excitation peak at ∼400 nm (Fig. 2b). Therefore, there is merit to sampling non-obvious residues with different aromatic nAAs to accelerate the generation of proteins with novel properties. Replacement of several residues with ioF or azF suppressed the minor 400 nm excitation peak and thus the population of the neutral chromophore in the ground state of sfGFP (ESI Fig. 2†). However, these changes were relatively minor in comparison to the variants that increase the proportion of the neutral form.

### The effects of aromatic nAA replacement at residue 44

**Fig. 3:**
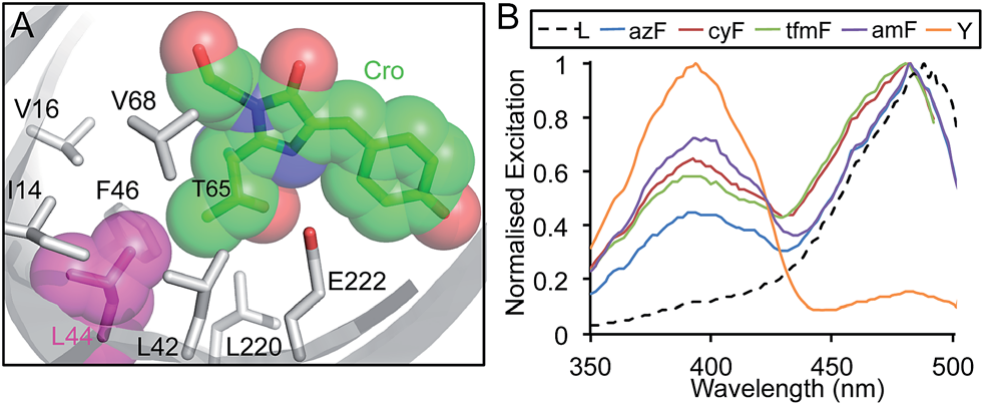
The influence of L44 mutations on sfGFP fluorescence. (a) Position of L44 (magenta) in sfGFP in relation to the chromophore (Cro; green) with neighbouring residues shown as sticks. PDB accession 2B3P. (b) Fluorescence excitation spectra of sfGFP with the indicated amino acid at position 44. Spectra were measured by monitoring emission at 511 nm on the soluble fraction of cell lysates from protein production cultures that were diluted to equivalent OD_600_ of 0.5.

It was clear that replacement of L44 with either ioF or azF produced the most significant changes in sfGFP fluorescence excitation of all the variants sampled. L44 has not to our knowledge been the target of traditional or nAA mutagenesis approaches to engineer the fluorescent properties of GFP and therefore the directed evolution approach has uncovered a novel mutation site. The L44 side chain is tightly packed in a largely hydrophobic core environment in the same plane as the chromophore (Fig. 3a). It packs close to the T65 moiety of the chromophore, at the opposite end to the phenol (contributed by Y66) group. The T65 moiety together with other nearby residues are known modulators of GFP fluorescence;^25,26^ small structural perturbations can have significant effects through disruption of charge-transfer networks that define the protonation state of the chromophore phenol group. Especially pertinent is the position of L44 parallel to E222 (Fig. 3a), the later being a pivotal residue in defining this polar bond network.^25,27,28^

Further investigation with additional aromatic nAAs demonstrated that changing the nature of the *para* substituent group modulated the excitation profile of the protein (Fig. 3). Three additional aromatic nAAs, *p*-cyano-l-phenylalanine (cyF), *p*-trifluoromethyl-l-phenylalanine (tfmF) and *p*-amino-l-phenylalanine (amF) (Fig. 1a), together with the natural analogue tyrosine were incorporated at residue 44. The different aromatic amino acids chosen have *para* substituents that vary in their polarity, electronegativity and hydrogen-bonding potential. All are factors known to influence the fluorescence properties of the chromophore.^24^ Furthermore, amF represents one of the potential photochemical endpoints for azF photolysis (*vide infra*).^9^ The effect of each aromatic nAA was to alter the ratio of neutral (*λ*_Ex_ ∼ 400 nm) to charged (*λ*_Ex_ ∼ 484 nm) forms of the chromophore in the ground state (Fig. 3b). The proportion of the neutral peak increased with the pattern Tyr > amF > cyF ≈ tfmF > azF > Leu. Y44 essentially forced the chromophore to adopt entirely the neutral form over the anionic. The amino (amF), cyano (cyF) and trifluoromethyl (tfmF) groups also promoted the neutral form but with the phenolate form dominating to different degrees depending on the aromatic nAA (neutral : anionic ratios of 0.7 : 1, 0.64 : 1 and 0.58 : 1, respectively). Additionally, all of the sampled aromatic nAAs resulted in a slight blue-shift in excitation (∼5 nm), which may be indicative of changes in residue packing and contacts around the chromophore.

### Photocontrol of sfGFP^L44azF^

Photoswitching autofluorescent proteins are of current interest as probes for super resolution imaging.^18^ Phenyl azide chemistry could provide a general route to genetically encode photocontrol in autofluorescent proteins (and proteins in general) with fast and large magnitude switching characteristics.^4,9^ Phenyl azides are known to be photochemically sensitive to light in the near UV and blue region and have classically been used as photocrosslinking reagents in biology through the generation of a reactive singlet nitrene radical on irradiation at physiologically relevant temperatures.^4,29–31^ Singlet nitrene is a highly reactive electrophile that can undergo a number of reactions including insertion into single bonds, addition to double bonds and reduction to a phenyl amine, which are environment dependant. Recent work from our lab using targeted mutagenesis has shown how different phenyl azide photochemical pathways can be used to modulate GFP fluorescence both *in vitro* and *in situ*, including by reduction to the phenyl amine (azF to amF) and addition reactions.^9^

Each of the sfGFP variants described above were produced containing azF and their light-dependent fluorescence was investigated. The orthogonality and fidelity of the tRNA-synthetase system used to incorporate azF is high, with the TAG codon reverting to a stop codon in the absence of azF from cell cultures (ESI Fig. 3;† ^9^). The parent sfGFP is largely insensitive to UV exposure of this kind,^9^ with the majority of aromatic nAA variants also displaying similar insensitivity (see P75azF as an example in ESI Fig. 4†). However, some variants displayed significant changes in their excitation and emission spectra on irradiation. Amongst these were the A37azF and L60azF variants, which exhibited a decrease in fluorescence emission intensity of between 35–40% on increasing exposure to UV (ESI Fig. 4†).

**Fig. 4:**
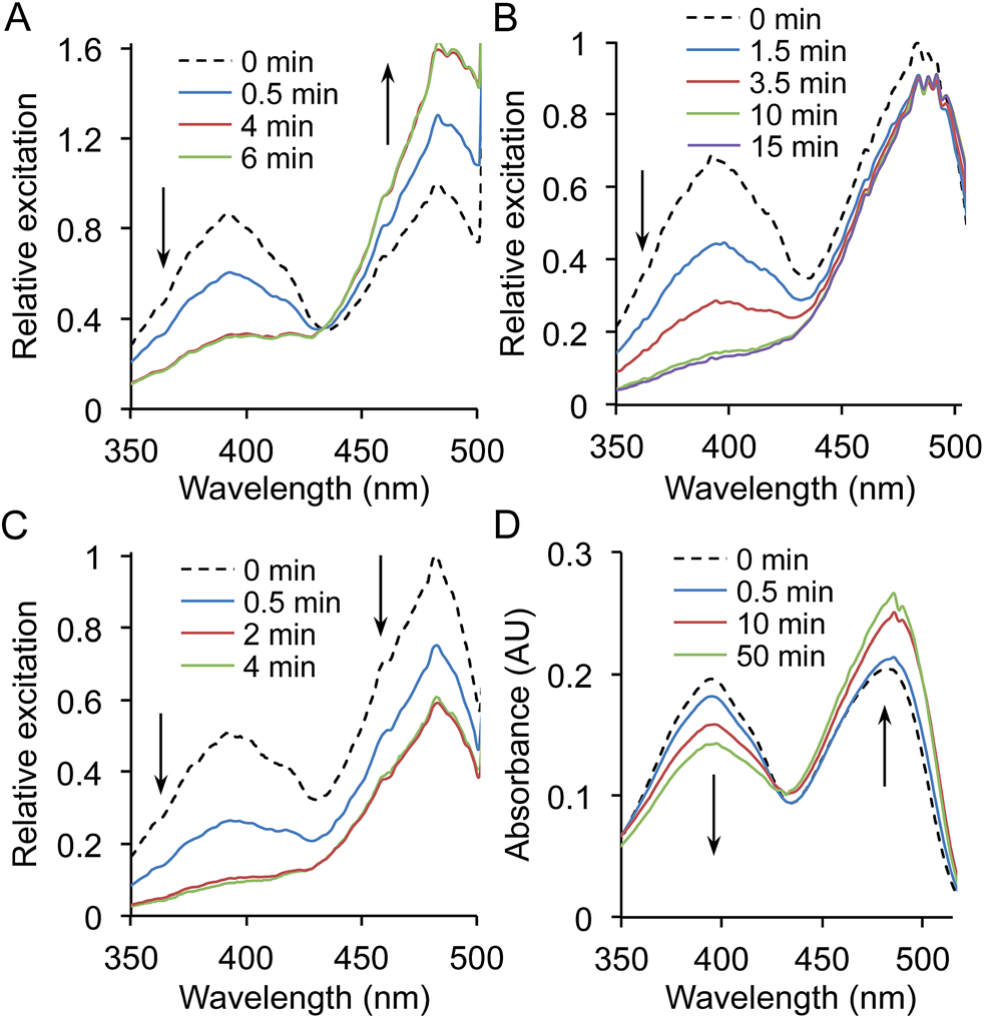
Photoswitching properties of sfGFP^L44azF^. Fluorescence photoswitching of sfGFP^L44azF^ was monitored on (a) whole cell, (b) cell lysate and (c) 1 μM pure protein samples. Samples in (a) and (b) were standardised to an OD_600_ of 0.5. Relative excitations was calculated by normalisation of fluorescence excitation intensity to 1 for the major 484 nm peak at time point 0 (no irradiation). Fluorescence excitation spectra were recorded following UV irradiation (302 nm, 6 W) for the indicated amounts of time by monitoring emission at 511 nm. Arrows indicate the change in fluorescence intensity over irradiation time. (d) Photoswitching behaviour of sfGFP^L44azF^ monitored by UV-vis absorbance. Spectra were recorded on 10 μM pure protein in phosphate buffer (100 mM, pH 8, 300 mM NaCl). The corresponding emission spectra are shown in ESI Fig. 5.†

The most notable effect was observed for L44azF (sfGFP^L44azF^). Production of the variant in the dark resulted in whole cell fluorescence excitation peak ratio (484 : 394 nm) of ∼1 : 1 (Fig. 4a and ESI Fig. 5a and b†). On irradiation the 394 nm excitation peak decreased and the 484 nm peak increased ratiometrically characterised by an isobestic point at 433 nm (Fig. 4a); the 394 : 484 nm excitation ratio stabilised at ∼1 : 5. As discussed above, the red shift in *λ*_Ex_ by 90 nm is indicative of a change in the ground state chromophore population from the neutral phenol to anionic phenolate.

The fluorescence changes of sfGFP^L44azF^ on photolysis appeared to be environmentally sensitive. In comparison to whole cell samples, a ratiometric response was not observed for lysed cells; the 394 nm excitation peak almost disappeared while the 484 nm peak remained constant (Fig. 4b and ESI Fig. 5c and d†). The change was different again for purified sfGFP^L44azF^, with irradiation resulting in a decrease in both excitation peaks and a near complete loss of the 394 nm peak (Fig. 4c and ESI Fig. 5e and f†). The bacterial cytosol is a complex mixture with proteins densely packed at a high effective concentration. Various properties such as ionic conditions, pH and redox potential are also regulated to maintain a constant intracellular environment. On preparation of cell lysate samples, cellular components are diluted and on protein purification are completely replaced by phosphate buffer. GFP is known to be sensitive to changes in pH and salt but both these had little effect on the photolysis characteristics apart from changing the initial fluorescence intensity (ESI Fig. 6 and 7†).

**Fig. 5:**
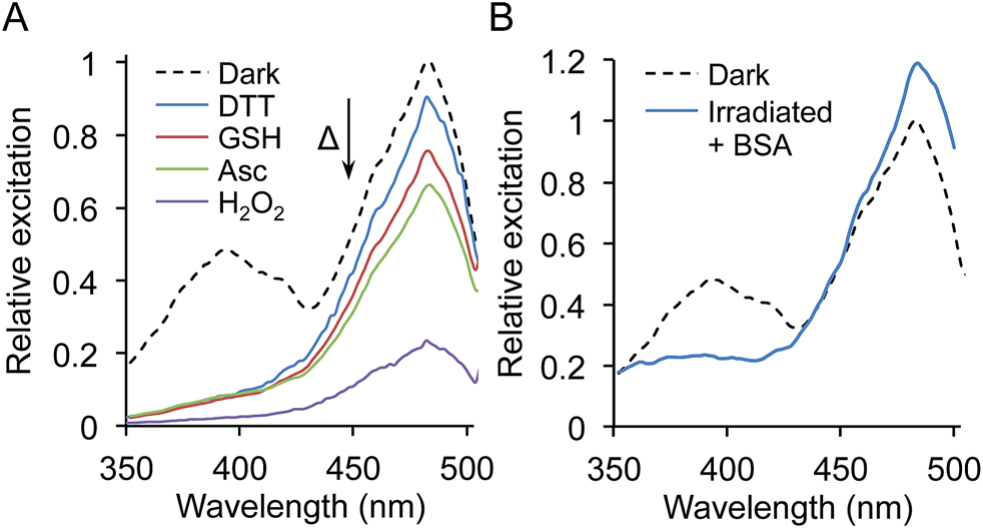
Redox and molecular crowding sensitive photoswitching of sfGFP^L44azF^. (a) Photoswitching of pure sfGFP^L44azF^ in the presence of different redox agents. The initial (‘dark’ reading) and final time points of irradiation for each redox agent are shown. (b) Photoswitching of sfGFP^L44azF^ in the presence of 200 mg mL^–1^ BSA showing the initial (‘dark’) and final time point of irradiation (60 min). Fluorescence excitation spectra are shown in (a) and (b) and were recorded on 1 μM sfGFP^L44azF^ by monitoring emission at 511 nm.

Phenyl azides can be sensitive to redox reagents, with one of the photochemical pathways being reduction to the phenyl amine, which can in turn influence GFP fluorescence.^9^ To test the effect of different redox agents on the photolysis characteristics of sfGFP^L44azF^, irradiation was performed with pure protein in buffers containing 1 mM dithiothreitol (DTT), reduced glutathione (GSH), ascorbate (Asc) or hydrogen peroxide (H_2_O_2_). While the general photolysis characteristics were similar to that in the absence of redox agent, the extent of the 485 nm peak decrease depended on the reducing agent used (Fig. 5a); stronger reducing agents giving rise to smaller proportional decreases at 485 nm compared to 400 nm with the pattern DTT > GSH > Asc (Fig. 5a). DTT gave a very similar photolysis pattern to that observed in cell lysates. The presence of the sole oxidizing agent, H_2_O_2_, was largely destructive with only ∼20% fluorescence intensity retained after irradiation (Fig. 5a). Incubation of the sfGFP^L44azF^ with each redox agent in the dark (no irradiation) had no effect on the excitation spectra profile, confirming requirement for irradiation to elicit the redox agent mediated effect.

Interestingly, under all conditions photolysis promoted conversion to the phenolate chromophore as suggested by increasing absorbance at 484 nm, with an isobestic point at ∼426 nm (Fig. 4d and ESI Fig. 8†). The different redox agents did affect the extent that the 484 nm peak increased on prolonged irradiation as represented by the differences in molar absorbance coefficients pre- and post-irradiation (ESI Table 3†); for example the presence of 1 mM DTT resulted in a 45% increase whereas H_2_O_2_ only increased by 23%. The blue-to-red absorbance shift on irradiation for sfGFP^L44azF^ suggests that the photochemical conversion is similar under the different redox conditions but the endpoint is redox sensitive, in terms of fluorescence emission. This is borne out in the changes in quantum yield (QY) on excitation at 484 nm. QY dropped from 0.69 to 0.51 on irradiation, tallying with the apparent drop in fluorescence emission intensity despite the increase in absorbance at 484 nm. In the presence of DTT the decrease in QY is less pronounced on irradiation (0.69 to 0.63). Combined, the data suggests that photolysis of the pure protein still promotes the anionic form of the chromophore over the neutral form but that the fluorescent capacity of the anionic form is reduced with a more significant non-radiative energy loss that is redox agent-dependent.

The initial loss of N_2_ from sfGFP^L44azF^ on photolysis could possibly lead to a rearrangement of the hydrogen bond network linked to the chromophore. The photochemical endpoint of the reactive nitrene thus may determine local fluorescence or, more accurately, quenching environment. We attempted to determine the photochemical endpoint by mass spectrometry but this was not feasible, as explained in the ESI.† One redox sensitive photochemical pathway is reduction of phenyl azide to a phenyl amine. This can be probed directly by incorporating *p*-amino-phenylalanine (amF; Fig. 1a) into sfGFP at residue 44 as in Fig. 3b. A peak at 394 nm corresponding to the neutral chromophore dominates the absorbance spectrum (ESI Fig. 9a†); the fluorescence excitation peak ratio (394 : 484 nm) is 0.7 : 1 (Fig. 3b and ESI Fig. 9b and c†). Both of these are dissimilar to the endpoint of sfGFP^L44azF^ irradiation suggesting that the phenyl amine is unlikely to be the photochemical endpoint. Despite our current limited understanding of the redox-dependent endpoint, these observed responses opens up the possibility of using sfGFP^L44azF^ as a genetically encoded cellular redox sensor for monitoring oxidative stress.

One condition that we were not able to fully replicate was high protein concentration. Intact *E. coli* cells experience high protein concentration, typically in the 1–10 mM range, and the cytosol is close to gel-like conditions. In an attempt to mimic intracellular conditions more closely, a high concentration (200 mg mL^–1^) of the commonly used inert protein BSA (bovine serum albumen) was added to 1 μM sfGFP^L44azF^ (in PBS) to act as a crowding agent. Subsequent photolysis resulted in the blue-to-red fluorescence switching characteristic of that observed for whole cell samples (Fig. 5b). However, no single isobestic point was observed as with whole cell samples. These results confirm the complexity of sfGFP^L44azF^ photoswitching but suggest a key role of molecular crowding.

### Understanding the structural impact of L44 mutations to aromatic nAA by molecular modelling

**Fig. 6:**
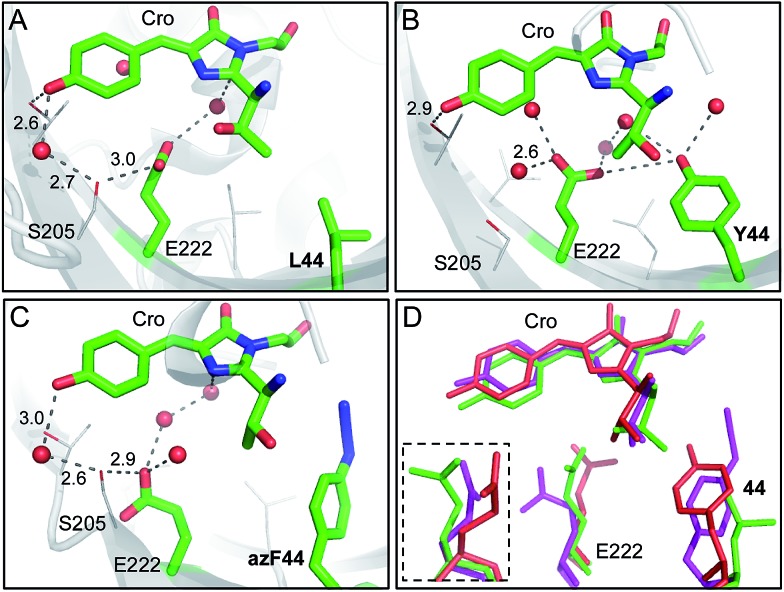
Molecular models of the sfGFP^L44^ aromatic nAA mutants. (a) X-ray crystal structure of the parent protein sfGFP (PDB accession ; 2B3P). Representative structural models of (b) sfGFP^L44Y^ and (c) sfGFP^L44azF^ obtained by molecular dynamics (50 ns). Residue 44, E222 and the chromophore (Cro) are shown as sticks and coloured by element with green carbon atoms in each case. Nearby residues are shown as lines and coloured by element with grey carbon atoms. Suggested hydrogen bonds are shown as black dashed lines and structural water molecules as red spheres with distance shown in Å. (d) Overlay of sfGFP (green), sfGFP^L44Y^ (red) and sfGFP^L44azF^ (pink) showing residues 44, E222 and the chromophore. The inset shows E222 from a different orientation (∼90° rotation).

The ability to model *in silico* the impact of mutations on proteins is a powerful way of matching phenotypic observations with underlying structural mechanisms, and form the basis for future protein design endeavours. There is currently limited *in silico* approaches^32^ to modelling and thus designing proteins containing nAAs. Here, we use a molecular dynamics approach to model the effect of mutating L44 to tyrosine or azF. The sfGFP^L44Y^ model suggests that a shift in the position of E222 side chain that directly impacts on the local hydrogen bonding network, including removing the indirect interaction through S205 to the phenol group of the chromophore (Fig. 6b). The model suggests that the side chain hydroxyl group of the T65 moiety forming the chromophore rotates away from E222 and the hydroxyl group of Y44 to form a new interaction with E222 both directly and *via* a structural water molecule. This new putative polar bond network is likely to stabilise the charged state of E222, which in turn prevents deprotonation of the chromophore phenol moiety by charge repulsion,^25^ as suggested by the experimental data (Fig. 3b).

The replacement of the *para* OH group with azido at residue 44 seemed to preclude either the wild type (L44) or Y44 arrangements and resulted in azF44 flipping away from E222 into a hydrophobic pocket (Fig. 6c). The consequence is that E222 shifts away from residue 44 whose side chain now occupies a position intermediate between sfGFP and sfGFP^L44Y^ (Fig. 6d). The shift in E222 does not appear to remove the key polar interaction with S205 but the position of S205 itself is shifted by ∼1.6 Å (Fig. 6). The consequence of this shift in S205 appears to be that the H-bond with the bridging water and the chromophore now lengthens by ∼0.4 Å and the chromophore loses the hydrogen bond with the OH group of Thr203. The intermediate position of E222 and its knock on effect on hydrogen bond network may correlate with excitation spectrum observed for sfGFP^L44azF^ suggesting the presence of both the charged and neutral form of the chromophore (Fig. 3b). Without knowing the endpoint product of sfGFP^L44azF^ it is difficult to build models concerning what might be occurring on photolysis. It is interesting to speculate that the loss of N_2_ will potentially provide more rotational freedom thus changing the relative position of the aromatic moiety, which in turn may influence the local polar network and thus chromophore charge.

### Aromatic nAA incorporation at T203 and photoswitching

**Fig. 7:**
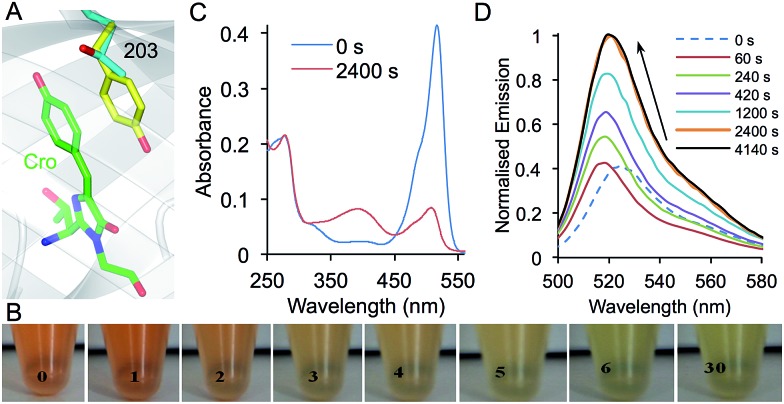
Photoswitching properties of sfGFP^T203azF^. (a) Structure of sfGFP showing the position of residue 203 in relation to the chromophore (Cro; green). Residue 203 is shown as the native Thr (grey) and Tyr as (yellow) as found in YFP (PDB accession ; 1YFP) demonstrating the π–π stacking interaction with the chromophore. (b) Ambient light photoswitching of sfGFP^T203azF^. Images of a cell lysate sample left in ambient room light for the indicated amount of time (in min). Photoswitching of sfGFP^T203azF^ monitored by (c) absorbance and (d) fluorescence emission. Photolysis was performed with a handheld UV lamp (302 nm, 6 W) for the indicated amount of time. Emission spectra were recorded on 1 μM protein after excitation at 485 nm and absorbance spectra with 10 μM protein.

Residue T203 lies directly above the plane of the chromophore (Fig. 7a) and has proved important in engineering the fluorescent properties of GFP. Replacement of T203 with natural aromatic residues red shifts *λ*_EM_ and *λ*_Ex_ through π–π stacking interactions with the chromophore and is the major mutation generating Yellow Fluorescent Protein (YFP; T203Y).^33,34^ This residue was not observed in the original library screening process but this may be due to its colour shift in fluorescence and its acute sensitivity to light (*vide infra*). A more likely explanation is that it may be a rarely occurring codon exchange mutation in the library^20^ so was not sampled during selection. Thus, residue 203 was the subject of targeted mutagenesis to assess the influence of aromatic nAAs. As expected, all of the aromatic nAAs sampled at residue 203 (azF, cyF, tfmF and amF) as well as tyrosine resulted in red-shifted fluorescence (ESI Fig. 10†) due to the predicted π stacking with the chromophore (Fig. 7a).

Given the proximity and intimate interaction between residue 203 and the chromophore, the introduction of azF at residue 203 should confer photosensitivity on sfGFP. sfGFP^T203azF^ was indeed very sensitive to irradiation with even ambient light eliciting a major change in the absorption properties and thus the transmitted light (Fig. 7b). In contrast to YFP, which confers a yellowish colour on *E. coli*, cells expressing sfGFP^T203azF^ were red in colour. The red transmittance properties suggest that the sfGFP^T203azF^ is absorbing in the blue, green and yellow region. On removal from the dark, the cell lysates changed from red to light green over the course of 30 min. More detailed studies on the purified protein carefully prepared to minimise light exposure revealed that sfGFP^T203azF^ was produced as a relatively poor fluorescent protein (compared to sfGFP) most likely due to the presence of the electron rich azido group, which can act as a quencher.^4,35^ Before irradiation, the sfGFP^T203azF^ had *λ*_Ex_ and *λ*_EM_ of 511 nm and 525 nm, respectively (*vide supra*). A minor excitation peak at ∼485 nm was also observed as a shoulder to the major 511 nm peak (ESI Fig. 11†). The absorbance spectrum showed an equivalent peak at 517 nm with an extinction coefficient of ∼41 400 M^–1^ cm^–1^ (Fig. 7c). On irradiation, the emission peak initially blue shifted by 8 nm and on further irradiation increased in intensity ∼3 fold and red shifted by 4 nm (to 521 nm; Fig. 7c). The rate of conversion to the endpoint was slower for the chromophore π-stacked phenyl azide at residue 203 than observed for sfGFP^L44azF^ (compare Fig. 4C and 7D). This is probably a function of the local microenvironment within the core of the protein. Interestingly, photoconversion of azF embedded within the highly delocalised chromophore (Y66azF mutation reported previously) with an extended conjugated system beyond the phenyl azide was equally as slow compared to positions that abut the chromophore.^9^ This suggests that photoreactivity of the phenyl azide could be influenced by the local electronic environment with electron donating and extended/interacting π systems slowing the rate.

Absorbance changes matched the shift in wavelength (517 to 509 nm), however the peak intensity dropped to ∼20% of the original with the emergence of a secondary peak at 393 nm suggesting the formation of a second non-radiative species (Fig. 7c). The increased fluorescence despite the drop in absorbance is evidence that a major change chemical environment of the chromophore occurs on photolysis. This could include a crosslink to the chromophore, which has been observed previously and is known to influence the electronic excitation properties (including loss of fluorescence) of the chromophore.^9^ The general red shift of the major excitation peak suggests that a significant population of photolysed sfGFP^T203azF^ retains the aromatic stacking configuration. The split population is unlikely to be due to the presence of neutral and phenolate chromophore states, as the blue absorbance peak is not fluorescent. The spectral properties of sfGFP^T203amF^ suggest that the final endpoint of sfGFP^T203azF^ photolysis is not the phenyl amine (ESI Fig. 9d and e†). Thus, it is clear that incorporation of azF at residue 203 instils highly sensitive photoswitching properties on sfGFP as well as red shifting its fluorescence. The photochemistry again appears to quite complex and would benefit for further structural and biophysical investigations. Further protein engineering or incorporation of additional photosensitive aromatic nAA may attenuate the sensitivity thus generating a potentially useful photoswitching autofluorescent protein.

## Conclusions

Here we have shown that aromatic nAAs with useful chemistry are tolerated within the β-barrel scaffold common to autofluorescent proteins, epitomised by sfGFP. This included within the core and at positions close to functional centres, despite the bulky nature and different chemical properties of the aromatic nAAs. The directed evolution approach unearthed residue positions not normally considered when engineering the fluorescence properties of GFP-based autofluorescent proteins. Phenyl azide chemistry was particularly useful as it instilled photoswitching properties not normally present in the parent protein when incorporated at particular residues. A key challenge lies in understanding how the protein environment defines the chemical route taken from the nitrene radical on photolysis.^36^ The use of *in silico* modelling with nAAs allows us to understand the impact of mutations outside the natural 20 amino acid set, which will lead the way towards *in silico* design of nAA containing proteins with useful and predictable properties.

## Experimental

Detailed experimental methods are provided in the ESI,† including TAG library construction, protein production, spectroscopy and molecular dynamic simulations.
