# Supplementary material for: Directed evolution of GFP with non-natural amino acids identifies residues for augmenting and photoswitching fluorescence
Source: Chem Sci. 2014 Nov 7;6(2):1159–66. doi: 10.1039/c4sc02827a (PMC5811120; doi:10.1039/c4sc02827a)
Supplement: Supplementary file 1 [file SC-006-C4SC02827A-s001.pdf]

Electronic Supplementary Material (ESI) for Chemical Science.
This journal is © The Royal Society of Chemistry 2014

Directed evolution of GFP with non-natural amino acids identifies residues for augmenting and photoswitching fluorescence

Samuel C. Reddington, Amy J. Baldwin, Rebecca Thompson, Andrea Brancale, Eric M. Tippmann & D. Dafydd Jones.

Supplementary Information

Generation of a TriNEx TAG library	2
General Methods	3-5
Mass spectrometry issues	5
Supplementary Figures 1-11 and Tables 1-3	7-15

Generation of a TriNEx TAG library

Initially, the engineered transposon MuDel (1) was randomly inserted into the pXP3 plasmid containing the *sfGFP* gene to create a library of 1.5×10^4 variants. *sfGFP* genes containing MuDel were then selected. MuDel excision then results in removal of a contiguous trinucleotide (Supp. Fig. S1). Replacement of the trinucleotide sequence with TAG was achieved using the DNA cassette $SubSeq^{TAG}$ as the donor of the TAG sequence (2). Two versions of the $SubSeq^{TAG}$ cassette were used to allow maximum sampling of TAG replacement irrespective of the orientation of $SubSeq^{TAG}$ insertion (Supp. Fig. S2). At this stage 2.9×10^4 variants were sampled. Removal of $SubSeq^{TAG}$ and the subsequent intramolecular ligation gave rise to $>1 \times 10^6$ colonies that formed the basis for selecting sfGFP variants with a single in-frame TAG codon placed randomly throughout the gene. At each stage, a suitable number of library members were deemed to be generated, so as to provide good coverage across the sfGFP gene (720 bp) and plasmid backbone (2.1 kbp).

Gene variants in the library containing in-frame TAG replacements were identified using a negative-positive cell-based selection as described previously (3). Library members conferring a fluorescent phenotype on *E. coli* in the absence of nAA were deemed not to contain an in-frame TAG as production of full length and fluorescent protein was most likely to have occurred. DNA was isolated from the $>1 \times 10^6$ colonies formed following the intramolecular ligation step and used to transform *E. coli* BL21 cells containing the nAA incorporation machinery plasmid (containing the tRNA/aminoacyl-tRNA-synthetase pair). Resulting non-fluorescent colonies (804) were selected and grown in the presence and absence of three different phenylalanine analogue nAAs with different *para* substituents (Fig. 1A): *p*-iodo-L-phenylalanine (ioF), *p*-azido-L-phenylalanine (azF) and *p*-acetyl-L-phenylalanine (acF). Approximately 10% of the colonies regained fluorescence in the presence of a nAA. Less than 1% of the colonies exhibited green fluorescence both in the absence and presence of nAA. Selected individual colonies with a fluorescent phenotype were subsequently re-grown on agar plates in the presence and absence of the nAA to remove any false positives from the final library (Supp. Fig. S1).

It should be noted that the aaRS engineered to incorporate ioF was used for tRNA charging in the presence of each utilised aromatic nAA due to the promiscuous activity of related aaRS towards different nAAs (3,(4). Furthermore, ioF was used in the initial variant selection phase. This may explain why on initial library analysis, more variants were discovered on selection with ioF. Despite this, a wide distribution of tolerated sites was observed (see main text).

General Methods

Generation of the T203stop mutant by rational site-directed mutagenesis

The amber stop codon mutation was introduced into the sfGFP gene resident within the pBAD/HisA plasmid (Invitrogen) using the Phusion® site-directed mutagenesis PCR protocol (New England Biolabs) as described previously (5) using the oligonucleotide primers T203TAG_F (5'-TAGCAGAGCGTTCTGAGCAAAGATCCG-3') and T203TAG_R (5'-GCTCAGATAATGATTATCCGGCAGCA-3'). Following PCR, linear DNA fragments were phosphorylated using T4 Polynucleotide Kinase (New England Biolabs) to allow recircularisation of the vector by Quick DNA Ligase (New England Biolabs). Mutants were used to transform *E. coli* TOP10 cells (Invitrogen), selected on LB agar containing ampicillin and sequence verified by the Cardiff University Molecular Biology Support Unit.

Production and purification of sfGFP variants

Wild-type sfGFP and the nAA containing variants were produced using a reprogrammed genetic code approach essentially as described previously (4, 5). *E. coli* TOP10 cells were co-transformed with both pBAD-sfGFP (or pBAD-sfGFP containing a TAG mutation) and pDULEaaRS which harbours the engineered *Methanocaldococcus jannascii* tyrosyl-tRNA-synthetase and $tRNA_{CUA}$ orthogonal pair required for nAA incorporation during cellular protein synthesis. Each nAA required a unique tRNA-synthetase and therefore pDULEaaRS plasmid; azF (pDULE-azF (6)), cyF (pDULE-cyF (7)), tfmF (pDULE-tfmF (8)), amF (pDULE-amF (6)) and Y (pDULE-wt-Y (7)). Single colonies grown on ampicillin and tetracycline LB agar were used to inoculate 5 mL LB broth cultures supplemented with the same antibiotics. After 16 hours of growth at 37 °C, the saturated cultures were used to inoculate ZYM-5052 arabinose autoinduction media (9) (1/200 dilution of the expression culture) supplemented with ampicillin and tetracycline. ZYM-5052 autoinduction media was composed of 1% (w/v) Tryptone, 0.5% (w/v) Yeast Extract, 0.5% (v/v) glycerol, 0.05% (w/v) glucose, 0.2% (w/v) lactose, 25 mM Na_2HPO_4, 25 mM KH_2PO_4, 50 mM NH_4Cl, 5 mM Na_2SO_4, 2 mM $MgSO_4$, trace metals and 0.05% (w/v) L-arabinose in ultra-pure water. The trace metal mixture contained 4 µM $CaCl_2$, 2 µM $MnCl_2$, 2 µM $ZnSO_4$, 0.4 µM $CoCl_2$, 0.4 µM $CuCl_2$, 0.4 µM $NiCl_2$, 0.4 µM Na_2MoO_4, 0.4 µM H_3BO_3, and 10 µM $FeCl_3$ in ultra-pure water. Cultures were grown at 37 °C with shaking for 1 hour before 1 mM of the required nAA was added. The cultures were then grown for a further 23 hours. Cells were harvested by centrifugation and lysed in sodium phosphate buffer (100 mM, 300 mM NaCl; pH 8)

containing 0.1 mg/mL lysozyme and 1 mM PMSF by French Press. Proteins were purified by Ni-affinity chromatography using HisTrap™ FF column (GE Life Sciences) attached to an ÄKTA Purifier. Proteins were further purified by anion exchange chromatography using a MonoQ™ column (GE Life Sciences) when required and buffer exchanged into 100 mM phosphate buffer, pH 8. Protein concentration was quantified using the D_C Protein Assay (BioRad).

Fluorescence spectroscopy

Excitation and emission spectra were recorded on variants using a Varian Cary Eclipse spectrophotometer at 20°C with a 5 × 5 mm QS quartz cuvette. Initially, the library was screened by measuring the fluorescence of whole cells suspended to an OD_{600} between 0.1 and 0.5. Two variants, G20 and V150, that conferred a weak fluorescent phenotype on cells grown on solid culture medium over 2 days did not produce any fluorescent sfGFP in the presence of either aromatic nAA in liquid cultures. All the remaining variants were fluorescent when expressed in the presence of ioF or azF.

Photolysis was performed in the cuvette using a 6 W UVM-57 mid-range UV handheld lamp (UVP) with a continuous spectrum from 275 – 380 nm and a peak emission at 310 nm. Samples were placed at a distance of 10 mm from the lamp and irradiated for various times, as indicated in the figures after which emission and excitation spectra were measured.

When more detailed analysis was performed, excitation and emission spectra were recorded on pure protein at 1 μM in PB (100 mM sodium phosphate, pH 8). Quantum yields were calculated using fluorescein (in 0.1 M NaOH) as a reference. Protein samples were diluted to a final absorbance of 0.05 at their respective λ_{max} and fluorescence emission spectra were recorded. Integrated emission intensity was calculated and used in the following formula to generate quantum yield values:

$$\phi_x = \phi_{st} . (Area_x/Area_{st}).(\eta^2{}_x/\eta^2{}_{st})$$

Where the ϕ_x and ϕ_{st} refer to the fluorescence quantum yield of the sample and fluorescein standard, respectively. $Area_x$ and $Area_{st}$ are the integrated emission intensities for the sample and fluorescein standard, respectively. η_x and η_{st} is the refractive index of the solvent for the sample and fluorescein standard, respectively. The refractive index correction here was negligible as 0.1 M NaOH and aqueous buffers differ in refractive index by <1%.

Absorbance spectroscopy

Absorbance spectra were recorded in PB at 20°C using either a Hewlett Packard diode array spectrophotometer (Agilent) or a JASCO V-660 spectrophotometer. Absorbance spectra were measured using 10 μM protein in a 1 cm pathlength QS quartz cuvette. Photolysis was performed as described above. Molar absorbance coefficients (ε) were calculated as outlined in Reddington *et al* (5) and Pedelacq *et al.*(10) Briefly, the UV-visible absorbance spectrum was measured before and after photolysis. Protein samples were then re-quantified using the Bio-Rad D_C assay to verify the concentration.

Molecular dynamic simulations

Molecular Dynamics simulations were performed using DESMOND 3.5 (11), via the Maestro interface (12), on a Viglen HPC computer equipped with a NVIDIA Tesla K20c GPGPU unit, running Ubuntu 12.04. The protein structures were prepared using Maestro (13), then they were soaked, neutralised and equilibrated using the DESMOND default scheme. The production dynamics where carried out for 50ns, in a NPT environment. Trajectory was analysed using Maestro. Resulting structures were compared to the crystal structure of the parent protein (L44; Fig. 6).

Mass spectroscopy issues.

We have attempted analysis of both the dark and photolysed variants by trypsin-digest MALDI- TOF with little success and have therefore included this more detailed description of experiments and issues.

Experimental procedure. The process was performed by the Central Biotechnology Services (CBS; Cardiff University) and involves excision of a protein sample from an SDS-PAGE gel. Proteins were destained with acetonitrile and ammonium bicarbonate. Samples were then dehydrated using acetonitrile and dried in an oven before reduction with 10 mM DTT at 56 °C. Samples were then alkylated with 55 mM iodoacetamide, washed and dehydrated again with acetonitrile and heating. Proteins were digested with sequencing grade modified trypsin (Promega) in ammonium bicarbonate. Peptides were spotted on 384 well MALDI target plates, mixed with matrix (α-cyano-4- hydroxycinnamic acid) and analysed by a 4800 MALDI TOF/TOF spectrometer (Applied Biosystems).

Analysis. The main problem with using any mass spec analysis for studying phenyl azide photochemistry is the similarity in mass of the potential photolysis products. The majority of products are within a couple of Da of each other with multiple possible products for a single mass and are thus near impossible to differentiate. It was for this reason that we chose trypsin-digest mass spec to decrease the fragment size for analysis and improve resolution.

The major issue that we experienced with this approach, which is inherent to the technique, is the partial detection of peptide fragments. Not all fragments fly and are detected with MALDI that unfortunately, for example, includes the fragment containing residue 44 (fragment LTLK; 474.34 Da). We have also tried trypsin-digest ESI-TOF in the past with no better results. To fully analyse the data we looked for peaks with the predicted mass for the mutations generated as well as shifts or the appearance of new mass peaks compared with the wild-type protein. Additionally we predict the mass of fragments crosslinked to peptides that would be in proximity to the azF in the protein. We have performed the experiments multiple times but have not seen evidence of any of the above.

Supplementary Figures 1-12 and Supplementary Tables 1-2

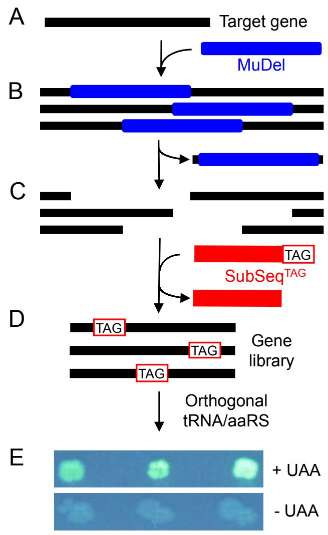

Supp. Fig. 1. Generation of TAG library and visual screen for in-frame TAG codons. The library is generated by the following steps: (A) transposon MuDel inserts randomly throughout the plasmid before *gfp*+MuDel is sub-cloned into pNOM-XP3 via NdeI and XhoI sites to select for 'in-gene' insertions; (B) MlyI digestion excises MuDel, also removing 3 bp, (C) which are then replaced by ligation of Subseq^{TAG}. Removal of Subseq using MlyI and the subsequent recircularisation generates a TAG replacement library (D); variants with in-frame TAG codons are identified by transformation of with the codon reprogramming plasmid and subjecting colonies to a negative (-UAA) followed by a positive (+UAA) selection process.

Supp. Table 1. Characteristics of variants tolerant to non-natural aromatic amino acid incorporation.

Residue	Codon change	azF	acF	ioF	2° structure	Surface accessibility (A^2)
P13	CCG→TAG	✔		✔	strand	64
V16	GTG→TAG	✔		✔	strand	0
L18	A-CTg→C-TAg	✔		✔	strand	2
G20	T-GGg→C-TAg	✔			strand	9
A37	T-GCg→C-TAg	✔		✔	helix	5
L44	CTG→TAG	✔	✔	✔	strand	7
P56	CCG→TAG			✔	core helix	9
W57	TGG→TAG	✔		✔	core helix	13
L60	CTG→TAG	✔	✔		core helix	0
P75	CCG→TAG	✔	✔	✔	helix	26
P89	CCG→TAG	✔		✔	loop	74
E95	GAA→TAG	✔		✔	strand	64
L119	CTG→TAG	✔	✔	✔	strand	0
R122	CGC→TAG		✔	✔	strand	55
S147	AGC→TAG		✔	✔	loop	62
V150	GTG→TAG			✔	strand	0
A154	CGC→TAG	✔	✔	✔	strand	42
L178	CTG→TAG	✔		✔	strand	48
P187	CCG→TAG	✔	✔	✔	strand	16
P192	CCG→TAG	✔		✔	loop	16
P196	CCG→TAG	✔		✔	loop	4
L201	T-CTg→C-TAg	✔		✔	strand	0
Q204	C-CAg→C-TAg		✔	✔	strand	73
P211	CCG→TAG	✔		✔	loop	130
F223	TTT→TAG	✔		✔	strand	81

Supp. Table 2. Fluorescence properties of non-natural amino acid substituted sfGFP variants. Wavelengths of excitation and emission are displayed relative to the parent sfGFP protein (in ± nm) shown with actual values in the top row. A value of 0 indicates no change compared to sfGFP. ND = not determined.

Residue	ioF (nm)			azF (nm)		
	1° λ_{ex}[a]	2° λ_{ex}[a]	λ_{em}[a]	1° λ_{ex}[a]	2° λ_{ex}[a]	λ_{em}[a]
Wild type	485	400	511	485	400	511
P13	+2		0	+1		0
V16	+1	Suppressed[b]	0	+1		0
L18	+1	Suppressed[b]	0	+1		0
G20	ND		ND	ND		ND
A37	+2		+1	+3		+1
L44	-7	-6 (1:0.4)[c]	-3	-2	-6 (1:0.32)[c]	-2
P56	-3		0	-5		-2
W57	+1		0	+1		0
L60	ND			-1		-1
P75	+7	0 (1:0.15)[c]	+1	+1		-2
P89	+3		0	+1		0
E95	+1		0	+1		0
L119	-6	Suppressed[b]	-2	+1		-2
R122	-2		0	+1		0
S147	-3		0	-3		0
V150	ND		ND	ND		ND
A154	+1		0	+1		0
L178	0		0	+1		0
P187	+3		0	+1		0
P192	0		0	+1		0
P196	+2			+1		0
L201	+8		+2	+5		0
Q204	+2	Suppressed[b]	0	+1	Suppressed[b]	-2
P211	-2	-5 (1:0.15)[c]	0	+3		0
F223	+2		0	+2		0

[a] the values for the variants as + or – nm values of that of the wild-type sfGFP. [b] 2° λ_{ex} at ~400 nm suppressed compared to sfGFP; [c] λ_{ex} of secondary peak with ratio of major:minor fluorescence in brackets (sfGFP 1:0.07).

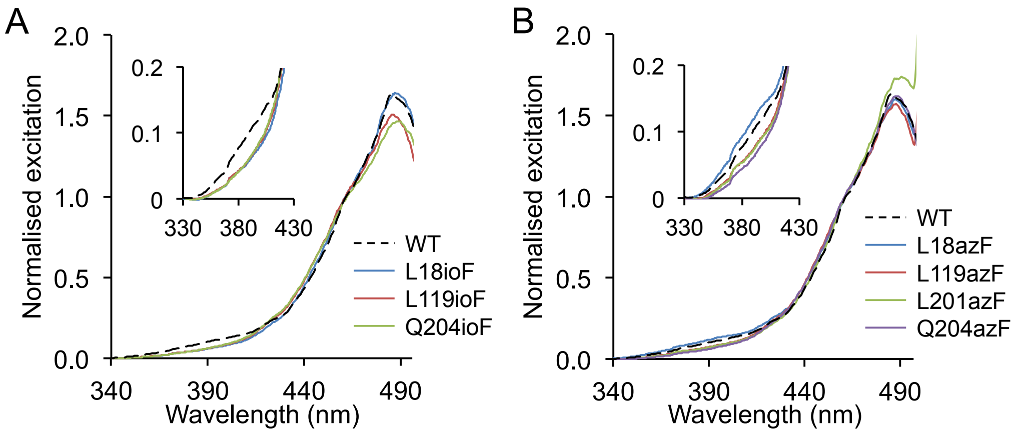

Supp. Fig. 2. Spectral properties of selected sfGFP^{nAA} variants. Fluorescence excitation spectra of (**A**) sfGFP^{ioF} and (**B**) sfGFP^{azF} variants with a suppressed secondary peak at ~400 nm. The inset shows a zoomed-in view of the secondary (neutral chromophore) excitation peak at ~400 nm. Spectra were recorded using cell lysates as source of variant (with an apparent OD_{600} of between 0.1 and 0.5) by monitoring emission at 511 nm. Spectra were normalised to a value of 1 at 460 nm. Three of these mutated residues (V16, L18 and L119) have side chains that stack in the core of sfGFP and point towards the T65 or G67 moieties of the chromophore. L201 is affected only by incorporation of azF and lies above the chromophore interacting with residues that directly modulate the properties of the chromophore (e.g. T203). Q204 lies immediately adjacent to the phenol group of the chromophore but with the side chain partially accessible to the solvent. Previous detailed analysis of the Q204azF sfGFP variant has shown that the extinction coefficient at 485 nm is higher than sfGFP (55,000 *vs* 49,000 $M^{-1}cm^{-1}$) (14). It is clear that introduction of ioF or azF at these positions is having a more direct effect on chromophore environment and further promoting the anionic form in the ground state.

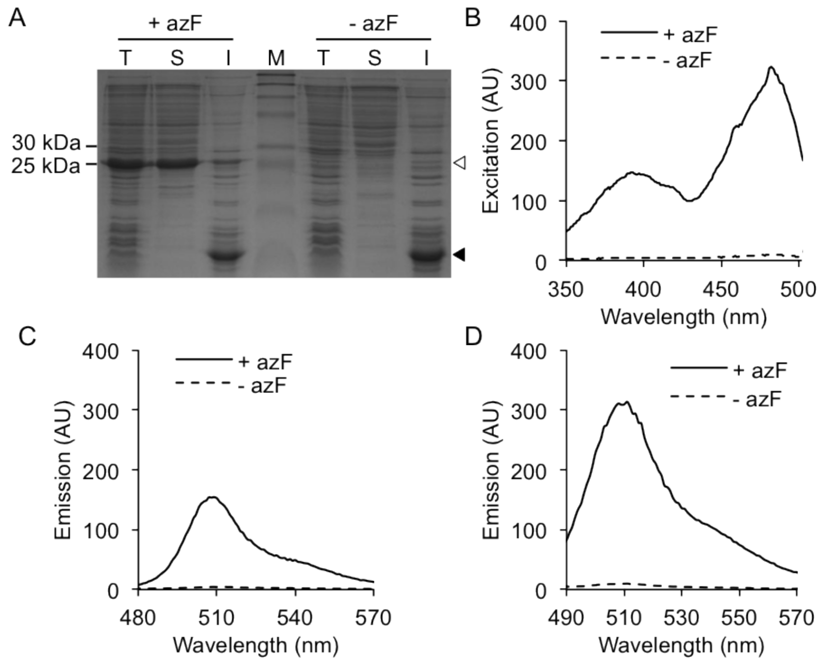

Supp. Fig. 3. Assessment of the orthogonal incorporation of azF into sfGFP. (**A**) SDS-PAGE analysis of cultures expressing sfGFPL44TAG grown in the presence (+ azF) or absence (- azF) of azF. Lanes refer to total cell contents (T), and soluble (S) and insoluble (I) fractions following cell lysis of expression cultures. The white arrow indicates the predicted full-length protein (27.8 kDa) and the black arrow indicates the predicted mass (~5 kDa) of sfGFP truncated at residue 44. (**B**) Fluorescence excitation (λ_{em} 511 nm), (**C**) emission (λ_{ex} 394 nm) and (**D**) excitation (λ_{ex} 484 nm) spectra of sfGFPL44azF cultures grown in the presence (solid) and absence (dashed) of azF. Cultures were lysed and soluble material was normalized to an effective OD_{600} = 0.5. Emission spectra were measured after excitation at the wavelength indicated in brackets (394 or 484 nm) and excitation spectra were measured by monitoring emission at 511 nm.

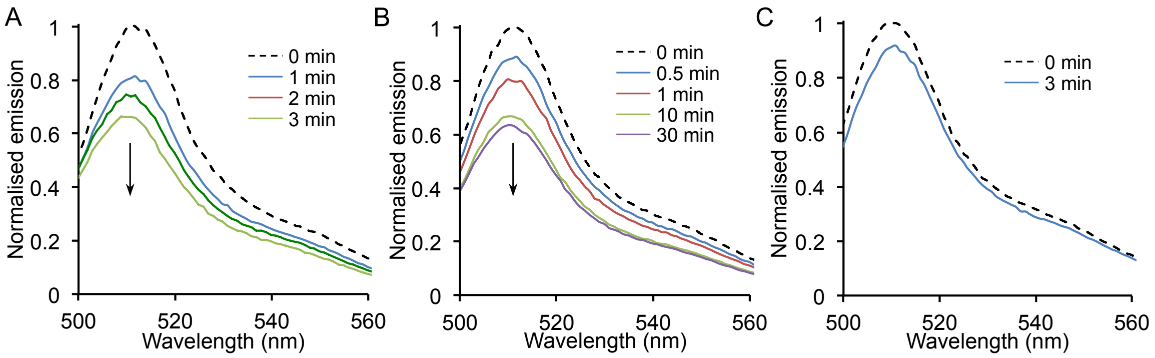

Supp. Fig. 4. Photoswitching properties of sfGFP^{azF} variants. Fluorescence emission spectra of (**A**) sfGFP^{A37azF}, (**B**) sfGFP^{L60azF} and (**C**) sfGFP^{P75azF} measured following irradiation (302 nm, 6W) for the indicated amount of time. Spectra were recorded on the soluble fraction of cell lysates (apparent OD_{600} of between 0.1 and 0.5) producing the sfGFP^{azF} variants after excitation at 485 nm. Each variant was produced in the dark to prevent any premature photoconversion and then irradiated with UV light (302 nm).

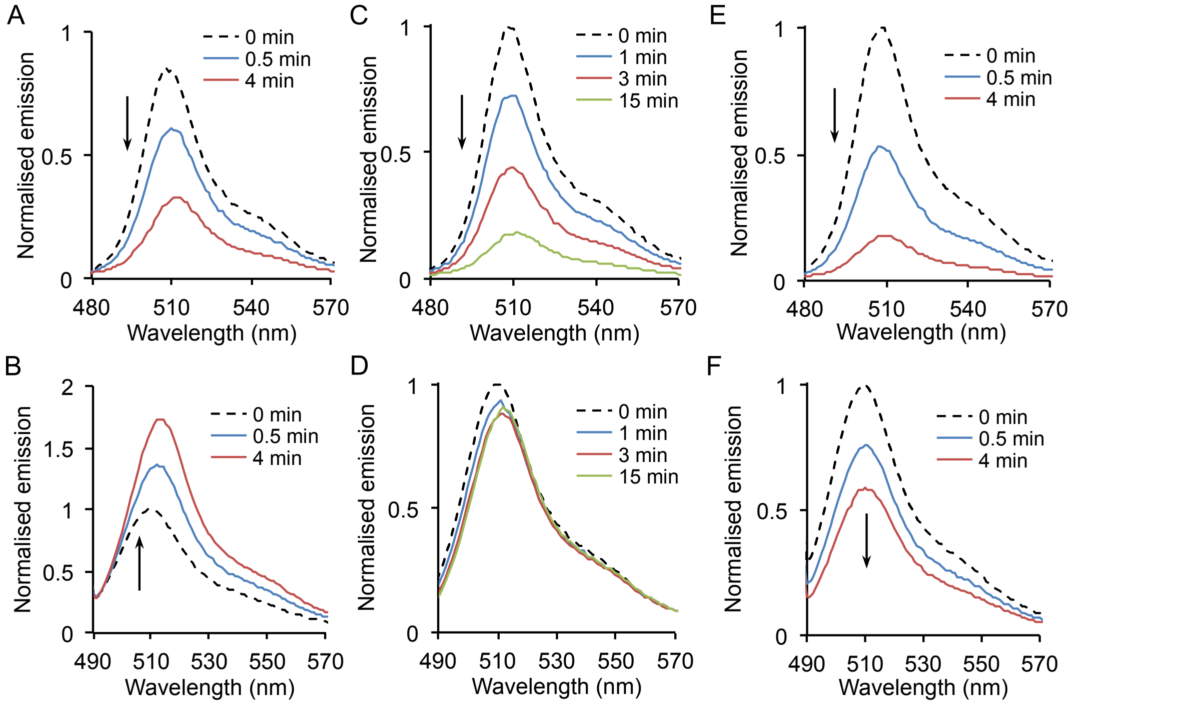

Supp. Fig. 5. Photoswitching properties of sfGFP^{L44azF}. Fluorescence photoswitching of sfGFP^{L44azF} was monitored on (**A**, **B**) whole cell, (**C**, **D**) cell lysate and (**E**, **F**) pure protein samples. Fluorescence emission spectra were recorded following UV irradiation (302 nm, 6 W) for the indicated amounts of time after excitation at 394 nm (top row) or 484 nm (bottom row). The pure protein spectra (**E**, **F**) were recorded on 1 μM protein in phosphate buffer (100 mM, pH 8). Arrows indicate the change in fluorescence intensity over irradiation time. The corresponding excitation spectra are shown in the main document Fig. 4.

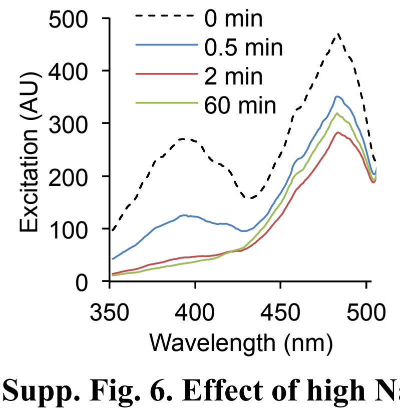

Supp. Fig. 6. Effect of high NaCl concentration on sfGFP^{L44azF} photoswitching. Fluorescence excitation spectra (λ_{em} = 511 nm) were recorded on 1 μM sfGFP^{L44azF} in 100 mM sodium phosphate buffer, pH 8, supplemented with 1 M NaCl. Irradiation was performed as in Supp Fig 3 and Fig. 4 for the indicated amount of time.

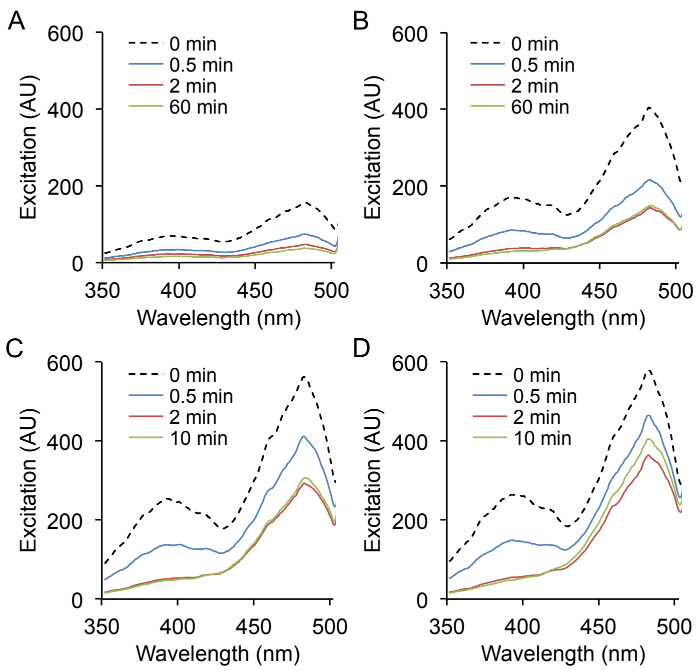

Supp. Fig. 7. Effect of pH on sfGFP^{L44azF} photoswitching. Fluorescence excitation spectra (λ_{em} = 511 nm) were recorded on 1 μM sfGFP^{L44azF} in 100 mM sodium phosphate buffered to a pH of (**A**) 5, (**B**) 6, (**C**) 7 or (**D**) 9. Spectra at pH 8 are shown in the main text. Irradiation was performed as in Supp Fig 3 Fig. 4 for the indicated amount of time.

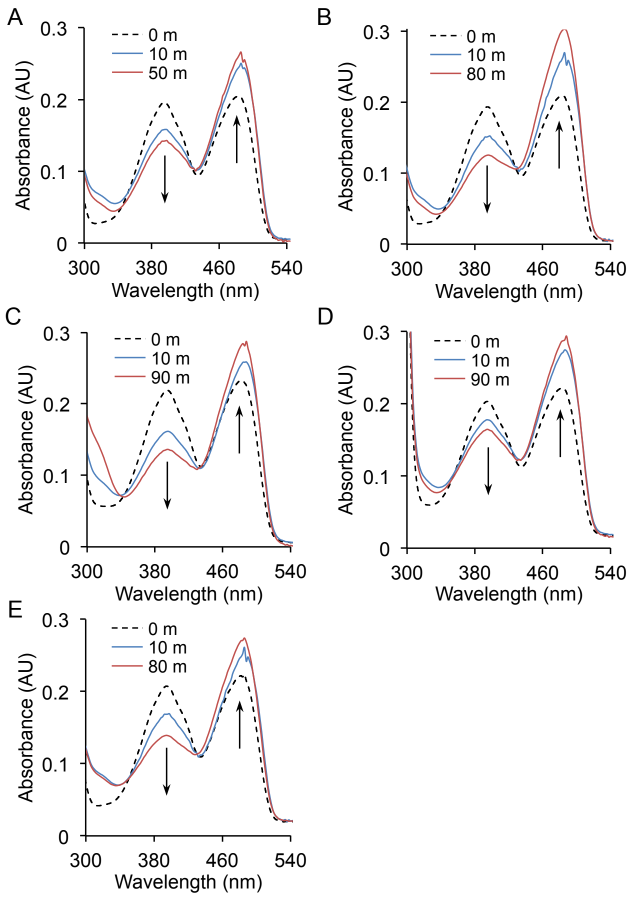

Supp. Fig. 8. Photoswitching behaviour of sfGFP^{L44azF} monitored by UV-vis absorbance. Spectra were recorded on 10 µM protein in phosphate buffer (100 mM, pH 8) with 1 mM of the following redox agent; (**A**) no additive, (**B**) DTT, (**C**) GSH, (**D**) ascorbate or (**E**) hydrogen peroxide.

Supp. Table 3. Spectral properties of sfGFP^{L44azF} (10 µM) before and after irradiation in the presence of different redox agents (1 mM).

Redox agent	Irradiation state	Extinction coefficient ($M^{-1}.cm^{-1}$)	
		λ_{394}	λ_{484}
None	Dark	19,600	20,500
None	Irradiated[a]	-27 %	+30 %
DTT	Irradiated	-35 %	+45 %
GSH	Irradiated	-37 %	+21 %
Asc	Irradiated	-24 %	+21 %
H_2O_2	Irradiated	-33 %	+23 %

[a] The percentage difference after irradiation was calculated from an initial 'dark' absorbance reading performed for each redox agent to avoid dilution error between conditions, using the equation: $\%_{change} = 100(A_{irrad}/A_{dark}) - 100$.

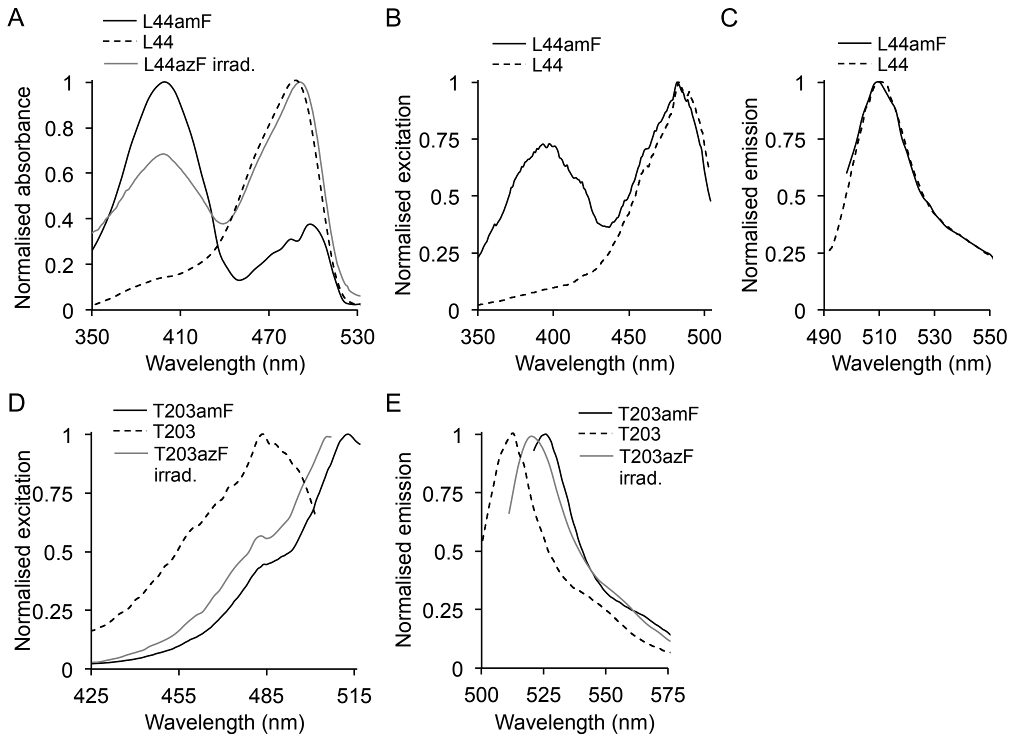

Supp. Fig. 9. Spectral properties of sfGFP variants containing the aromatic nAA amF. (**A**) Fluorescence excitation (λ_{em} = 511 nm) and (**B**) emission (λ_{ex} = 484 nm) spectra of sfGFP^{L44amF} recorded on 1 μM pure protein in PBS (100 mM sodium phosphate, pH 8, 300 mM NaCl). The spectra (solid lines) are overlaid with that of wild-type sfGFP (dashed lines) for comparison and intensities normalised to 1 at their λ_{max}. (**C**) Absorbance spectrum of sfGFP^{L44amF} (solid, black line) in comparison to the wild-type sfGFP (dashed line) and the irradiated form of sfGFP^{L44azF} (grey line; PBS, no additive). (**D**) Fluorescence excitation (λ_{em} = 525 nm) and (**E**) emission (λ_{ex} = 513 nm) spectra of sfGFP^{T203amF}. The spectra (solid lines) are overlaid with that of wild-type sfGFP (dashed lines) and the irradiated form of sfGFP^{T203azF} (grey line) for comparison with intensities normalised to 1 at their λ_{max}.

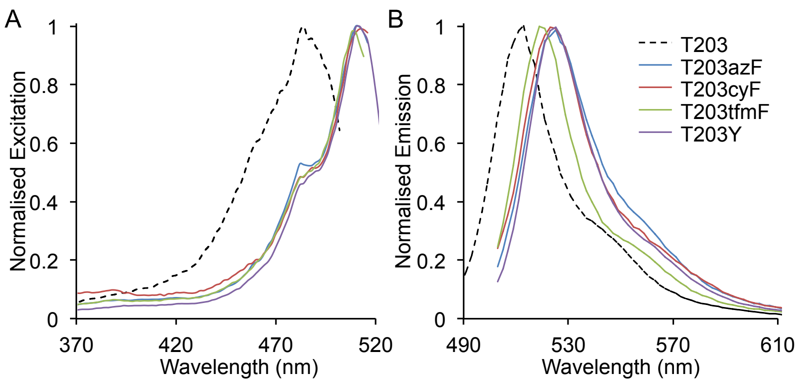

Supp. Fig. 10. Spectral properties of sfGFP variants containing aromatic nAAs at residue 203. (**A**) Excitation and (**B**) emission spectra of sfGFP variants containing the indicated aromatic nAA at residue 203. The parent protein (T203; dashed line) is included for comparison. Emission spectra were recorded following excitation of the sample at the respective λ_{EX} and excitation spectra by monitoring emission at the λ_{EM}. Spectra were measured on soluble cell lysates from protein production cultures that were diluted to apparent OD_{600} between 0.1 – 0.5. The λ_{EX} were red-shifted by 23 nm (to 508 nm) for tfmF, 26 nm (to 511 nm) for azF, cyF, and Y, and 28 nm (to 513 nm) for amF compared to sfGFP. The emission profile matched the red-shifted pattern observed for the excitation with λ_{EM} red shifted to 520 nm for tfmF, 525 nm for azF, cyF and Y, and 526 nm for amF. The small variations in λ_{EX} and λ_{EM} can be explained by the electron donating or withdrawing abilities of the *para* ring substituents with more electron donating groups resulting in further red-shifts. The trifluoromethyl group is the most electron withdrawing, therefore reducing the electron density delocalized with the stacked chromophore and hence $sfGFP^{T203tfmF}$ has the least red-shifted fluorescence. The azido (azF) and cyano (cyF) groups contain delocalized bonds so should act as mild electron withdrawers and show intermediate λ_{EX} and λ_{EM}. The amino group (amF) is the most electron donating and indeed $sfGFP^{T203amF}$ shows the furthest red-shift in fluorescence.

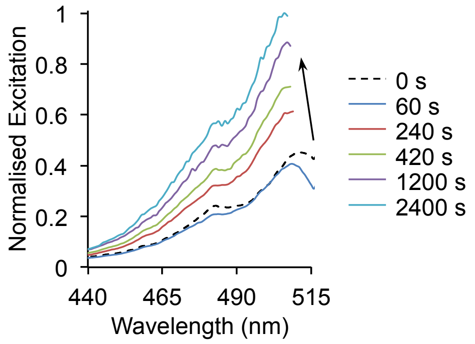

Supp. Fig. 11. Photoswitching behaviour of $sfGFP^{T203azF}$. Fluorescence excitation spectra of $sfGFP^{T203azF}$ measured after irradiation (302 nm, 6 W) of the purified protein (1 μM) for the indicated amount of time. Spectra were recorded by monitoring emission at 525 nm.

References

1. Jones DD. Triplet nucleotide removal at random positions in a target gene: the tolerance of TEM-1 beta-lactamase to an amino acid deletion. Nucleic acids research. 2005;33(9).
2. Baldwin A, Busse K, Simm A, Jones D. Expanded molecular diversity generation during directed evolution by trinucleotide exchange (TriNEx). Nucleic acids research. 2008;36(13):77-86.
3. Baldwin A, Arpino J, Edwards W, Tippmann E, Jones D. Expanded chemical diversity sampling through whole protein evolution. Molecular BioSystems. 2009;5(7):764-6.
4. Miyake-Stoner SJ, Refakis CA, Hammill JT, Lusic H, Hazen JL, Deiters A, et al. Generating permissive site-specific unnatural aminoacyl-tRNA synthetases. Biochemistry. 2010;49(8):1667-77.
5. Reddington SC, Tippmann EM, Jones DD. Residue choice defines efficiency and influence of bioorthogonal protein modification via genetically encoded strain promoted Click chemistry. Chemical communications. 2012.
6. Reddington SC, Rizkallah PJ, Watson PD, Pearson R, Tippmann EM, Jones DD. Different Photochemical Events of a Genetically Encoded Phenyl Azide Define and Modulate GFP Fluorescence. Angewandte Chemie International Edition. 2013;52.
7. Miyake-Stoner SJ, Miller AM, Hammill JT, Peeler JC, Hess KR, Mehl RA, et al. Probing Protein Folding Using Site-Specifically Encoded Unnatural Amino Acids as FRET Donors with Tryptophan. Biochemistry. 2009;48(25):5953-62.
8. Hammill JT, Miyake-Stoner S, Hazen JL, Jackson JC, Mehl RA. Preparation of site-specifically labeled fluorinated proteins for 19F-NMR structural characterization. Nature protocols. 2007;2(10):2601-7.
9. Studier FW. Protein production by auto-induction in high-density shaking cultures. Protein expression and purification. 2005;41(1):207-34.
10. Pedelacq J, Cabantous S, Tran T, Terwilliger T, Waldo G. Engineering and characterization of a superfolder green fluorescent protein. Nature biotechnology. 2005;24(1):79-88.
11. Bowers KJ, Chow E, Xu H, Dror RO, Eastwood MP, Gregersen BA, et al., editors. Scalable algorithms for molecular dynamics simulations on commodity clusters. SC 2006 Conference, Proceedings of the ACM/IEEE; 2006: IEEE.
12. Desmond Molecular Dynamics System v, D. E. Shaw Research, New York, NY, 2013. Maestro-Desmond Interoperability Tools, version 3.5, Schrödinger, New York, NY, 2013.
13. Schrödinger Release 2013-2: Maestro v, Schrödinger, LLC, New York, NY, 2013. http://www.schrodinger.com.
14. Reddington SC, Tippmann EM, Jones DD. Residue choice defines efficiency and influence of bioorthogonal protein modification via genetically encoded strain promoted Click chemistry. Chemical communications. 2012;48(67):8419-21.
